# Clustering of host-seeking activity of *Anopheles gambiae* mosquitoes at the top surface of a human-baited bed net

**DOI:** 10.1186/1475-2875-12-267

**Published:** 2013-07-31

**Authors:** Amy Lynd, Philip J McCall

**Affiliations:** 1 Liverpool School of Tropical Medicine, Pembroke Place, Liverpool, L3 5QA, UK

**Keywords:** Bed net, LLIN, ITN, Anopheles gambiae, Two-in-one, Vector behaviour, Mosquito, Malaria, Pyrethroid, Insecticide resistance

## Abstract

**Background:**

Knowledge of the interactions between mosquitoes and humans, and how vector control interventions affect them, is sparse. A study exploring host-seeking behaviour at a human-occupied bed net, a key event in such interactions, is reported here.

**Methods:**

Host-seeking female *Anopheles gambiae* activity was studied using a human-baited ‘sticky-net’ (a bed net without insecticide, coated with non-setting adhesive) to trap mosquitoes. The numbers and distribution of mosquitoes captured on each surface of the bed net were recorded and analysed using non-parametric statistical methods and random effects regression analysis. To confirm sticky-net reliability, the experiment was repeated using a pitched sticky-net (tilted sides converging at apex, i.e., neither horizontal nor vertical). The capture efficiency of horizontal and vertical sticky surfaces were compared, and the potential repellency of the adhesive was investigated.

**Results:**

In a semi-field experiment, more mosquitoes were caught on the top (74-87%) than on the sides of the net (p < 0.001). In laboratory experiments, more mosquitoes were caught on the top than on the sides in human-baited tests (p < 0.001), significantly different to unbaited controls (p < 0.001) where most mosquitoes were on the sides (p = 0.047). In both experiments, approximately 70% of mosquitoes captured on the top surface were clustered within a 90 × 90 cm (or lesser) area directly above the head and chest (p < 0.001). In pitched net tests, similar clustering occurred over the sleeper’s head and chest in baited tests only (p < 0.001). Capture rates at horizontal and vertical surfaces were not significantly different and the sticky-net was not repellent.

**Conclusion:**

This study demonstrated that *An. gambiae* activity occurs predominantly within a limited area of the top surface of bed nets. The results provide support for the two-in-one bed net design for managing pyrethroid-resistant vector populations. Further exploration of vector behaviour at the bed net interface could contribute to additional improvements in insecticide-treated bed net design or the development of novel vector control tools.

## Background

The insecticide-treated bed net (ITN) is one of the most effective tools available for the prevention of malaria, protecting those who sleep underneath and when coverage is high, the greater community, including those without nets [1,2]. With sustained high coverage, ITNs can reduce uncomplicated malaria by half, leading to increased haemoglobin levels and marked reductions in all cause child mortality [1]. The current generation of bed nets, termed long-lasting insecticidal nets (LLINs), remain central to malaria control and elimination in Africa where indoor transmission of malaria is of major importance [3]. However, resistance to pyrethroids, the only class of insecticides approved for use on LLINs, is emerging at an alarming rate in *Anopheles gambiae sensu stricto (s.s.)*, the main indoor-biting vector of malaria in Africa and the species most effectively targeted by LLINs [4-7], threatening malaria control [8]. If LLINs are to remain central to malaria prevention, then new designs or approaches are urgently needed.

One solution already proposed is the combination/mosaic net or ‘two-in-one’ net, where insecticides, synergists or repellents are combined to maximize the lifespan of the pyrethroid. Initial trials have indicated that such two-in-one nets can be effective against pyrethroid-resistant *An. gambiae*[9-14], but how these or other novel LLIN treatments perform in the long term will depend on how vector mosquitoes interact with nets as they try to reach the hosts within. Predicting this is uncertain since little is known about mosquito activity at the interface of a human-occupied bed net. It has been proposed that bed nets lure mosquitoes to the top of the net [9]. Though plausible, there is no confirmed experimental evidence that this is true.

To better understand mosquito behaviour around a sleeping host, the findings of a study to map the activity patterns of host-seeking female *An. gambiae* at a human-baited bed net are reported here*.*

## Methods

### Mosquitoes

Mated unfed adult female (aged three to seven days post-eclosion) *An. gambiae s s*, Kisumu strain were obtained from colonies maintained at LSTM, and at CDC/KEMRI in Kisian, Kenya. Colonies were maintained according to standard protocol. Mosquitoes at CDC/KEMRI were routinely fed on restrained rabbits, whilst at LSTM colonies were maintained using artificial membranes and human blood.

### The sticky-net

Experiments measured the location of mosquito landings on an untreated (without insecticide), baited (with a human inside) bed net. The bed net was supported on a wooden frame over a single raised bed and a grid was drawn on each surface in indelible pen before coating the entire outer surface with a non-setting adhesive (‘Tangle-Trap’ Liquid Insect Trap Coating, The Tanglefoot Company, MI, USA) of low toxicity and odour, viscosity, persistence, and apparent low repellency to mosquitoes (see results of repellency experiments). Three coats of adhesive were applied at 24-hour intervals to ensure complete and adequate coverage without obstructing the net mesh, and each net was re-used until damaged.

### Experimental procedures of all sticky net trials

With a sleeper under the sticky-net, 100 female *An. gambiae* (aged three to seven days post-eclosion) within a holding pot were placed in the experimental tent/room for 30 min before release. Other than orientation, volunteer’s position (lying on back, side, or stomach) was not prescribed. After each experiment, the locations of caught mosquitoes were recorded and the remaining free-flying mosquitoes killed.

### Semi-field tests with a human-baited sticky-net

**Figure 1:**
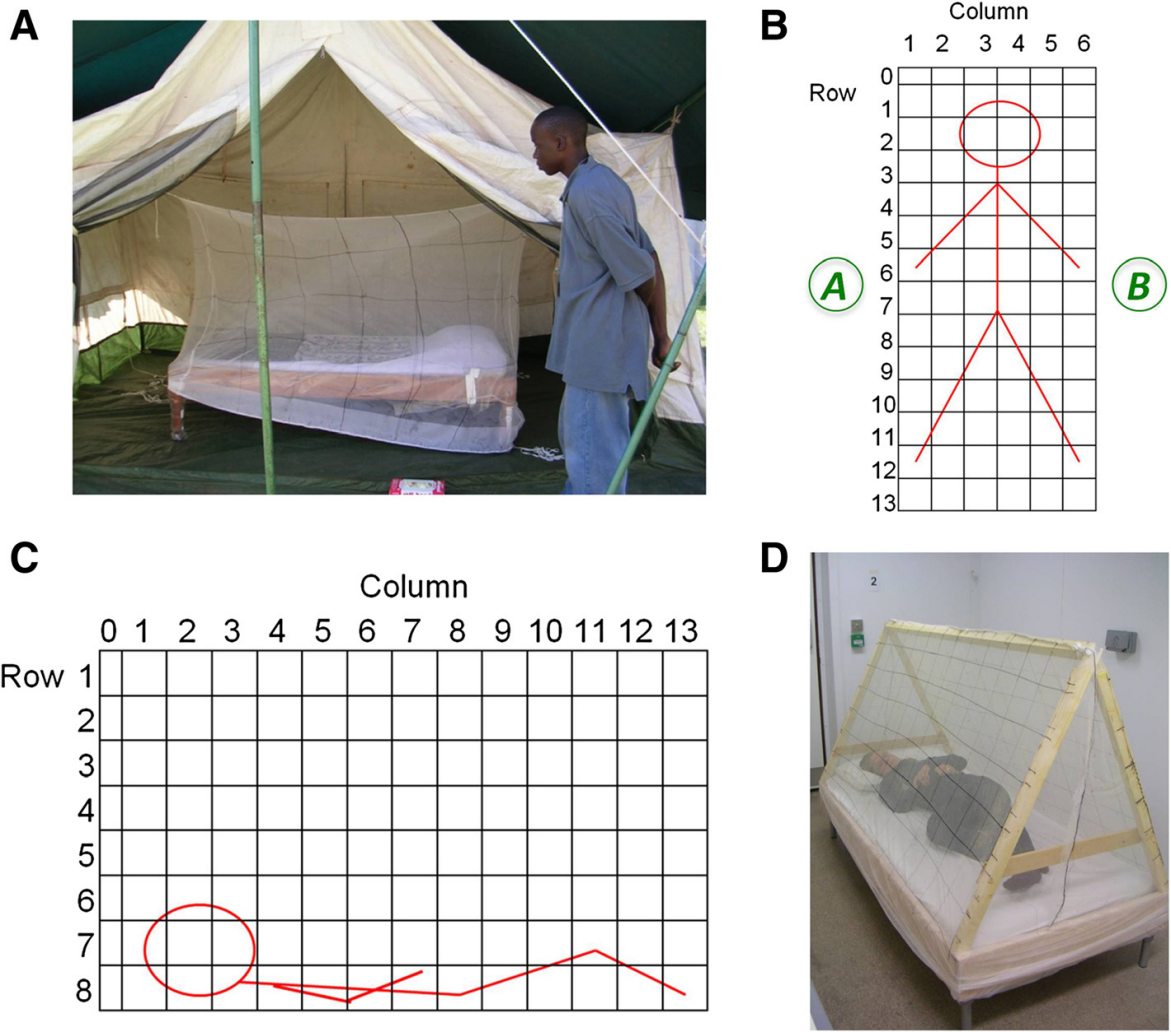
**Experimental set-up of semi-field and laboratory sticky-net trials. A**. The set-up used in semi-field trials showing the bed and sticky-net (with 30 sq cm grid marking) *in situ* inside the canvas tent. **B**. Diagram of the grid scheme on the top surface of the standard rectangular used in laboratory sticky-net trials, and the position of the human bait beneath. Grid squares were 15 sq cm, except row 0 which were 7.5 × 15 cm. Positions of the head/feet were reversed in each of two repeat trials for each volunteer. Mosquitoes were released at positions **A** or **B**, and the release container was located either on the floor or at the ceiling. **C**. Diagram of the grid numbering scheme on the side surfaces of the pitched net in relation to the human bait within. Grid squares were 15 sq cm, except row 0 which were 7.5 × 15 cm. **D**. The pitched sticky-net in the climate controlled room with a human bait inside.

The initial study was undertaken at CDC/KEMRI in Kisian, Kenya. Colony-reared mosquitoes were released inside a large canvas tent (3.5 × 2.5 × 2.5 m high at apex) containing the rectangular baited sticky-net (locally made untreated polyester bed net, 2.1 × 0.9 × 1.5 m high), marked with a 30 sq cm grid (Figure 1A).

Mosquitoes were released from at ground level from position A or B (Figure 1B) as previously described. Three experiments using the same volunteer were undertaken between 21.00 and 06.30 on consecutive nights. Sleeper orientation was reversed on each night. A second volunteer waited in a neighbouring shelter (approximately 20 meters away) throughout the experiment, to provide assistance if needed.

### Laboratory tests with a human-baited sticky-net

Following the semi-field trial, a series of laboratory trials using multiple sleepers was undertaken. This and all subsequent experiments were carried out in the dark in a climate-controlled room (4.7 × 2.8 × 2.3 m high; 25–27°C, 65–95% RH) in Liverpool. Rectangular sticky-nets (2.05 × 0.9 × 1.0 m high; 100-denier untreated polyester bed net; Siam Dutch Mosquito Netting, Thailand), marked with a 15 sq cm grid (Figure 1B) were used with seven adult human volunteers of both sexes, various ages and ethnicity. Each person was tested twice (with head-feet position reversed) in experiments of four hours’ duration; four unbaited control experiments were also carried out. Mosquitoes were released from position ‘A’ or ‘B’ (Figure 1B), either at ground level or from close to the ceiling. On their first trial, each volunteer tested was assigned a randomly selected combination of head/foot orientation, mosquito release location and release height. On their second trial, the alternative position for each variable was used. Thus each person was tested in both orientations, against both mosquito release sides and heights, and, when possible, an equal number of tests of all combinations and variables were carried out.

### Reliability and accuracy of the sticky-net

The possibility that the top horizontal surface might have been more efficient at trapping mosquitoes than the vertical sides was investigated in two additional experiments. A third experiment measured potential repellency of the adhesive treatment.

### Laboratory tests with a human-baited pitched sticky-net

To eliminate orientation bias, laboratory experiments were repeated using a pitched or tent-shaped bed net. A purpose-made sticky-net of triangular cross-section (2.05 × 1.10 m on each vertical side; total height 1.00 m) was placed over the bed base and marked with a 15 sq cm grid (Figures 1C and 1D). The experiment was carried out using eight adult volunteers (16 experiments) and eight unbaited controls, as described previously for the standard bed net study.

### Direct observation of the effect of sticky-net surface orientation

**Figure 2:**
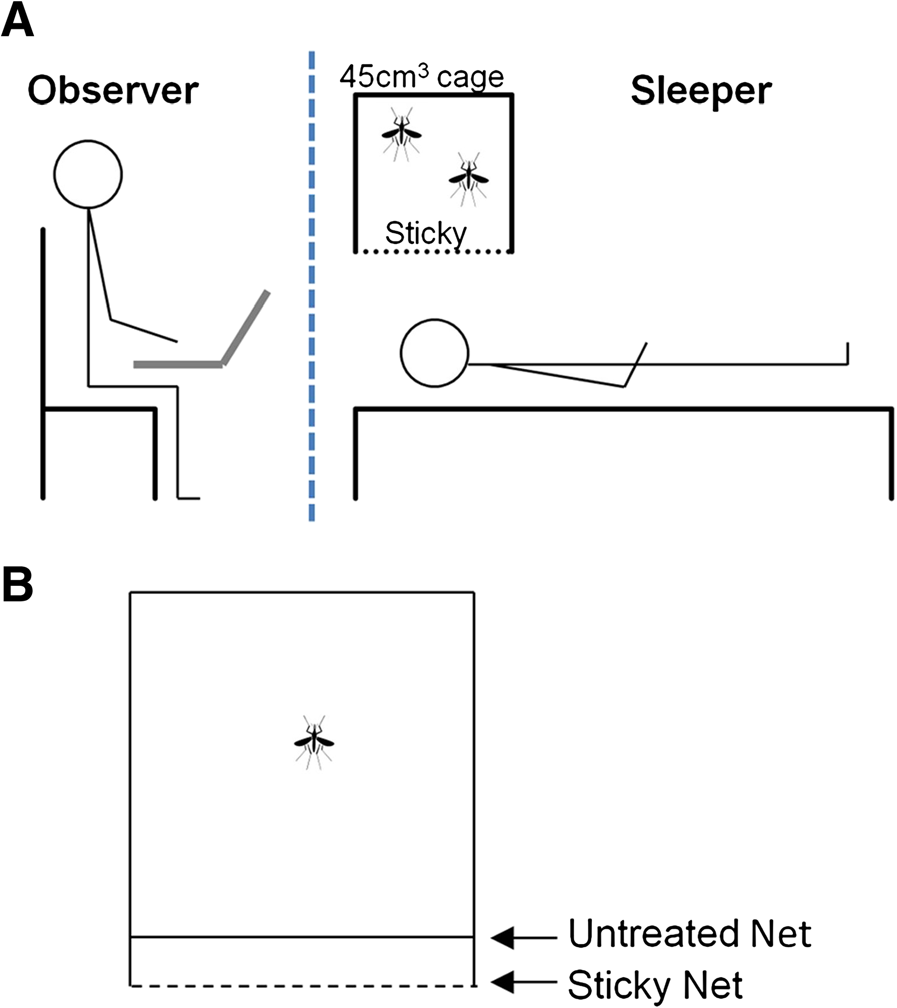
**Experimental set-up for direct observation tests. A**. Set-up used to assess catch rates at horizontal and vertical sticky-net surfaces - only the horizontal experimental set-up is shown. The dashed blue line represents the clear Perspex screen (200 × 150 cm) separating the observer from the experimental set-up. **B**. Detail of modification used to investigate potential non-contact repellency of the sticky-net treatment. The distance between the two net surfaces was 2 cm. In control trials, two layers of untreated net were used.

A second investigation into possible differences in trapping efficiency by horizontal and vertical nets was carried out by direct observation of mosquitoes at a sticky surface (Figure 2), in an insectary with minimal lighting. One surface of a 45 sq cm cage was replaced with sticky-netting. The cage was suspended 50 cm above (presenting a horizontal surface to the test mosquitoes; Figure 2A) or to one side (presenting a vertical surface to the test mosquitoes) of the face of a prone human. A large acrylic screen (2.0 × 1.5 m) was placed between the observer and the experimental set-up as a barrier to potential attractants from the observer.

In the first test, 20 *An. gambiae* were released remotely from the holding chamber into the cage, and the numbers of visits (V - contact with the net that did not result in a stick event), sticks (S - mosquito visibly held by the sticky-net), and escapes (E - mosquito escaped after being held temporarily) over a 30-min period were recorded using Noldus Observer 5.0 event recorder software (Noldus Information Technology, Wageningen, The Netherlands). The numbers permanently ‘captured’ (S-E), and total number of ‘contacts’ regardless of outcome (S + V) were calculated. A total of ten experimental replicates were carried out: five each at horizontal and vertical surfaces.

The second test observed 72 female mosquitoes performing individually, under the same conditions. The experiment proceeded for 30 min or until the mosquito was captured (defined as stuck on the net for 5 min). If the mosquito stopped visiting the net for a period of 10 min at any time during the experiment, the data were discarded.

### Repellency of the adhesive treatment

The possibility that the adhesive treatment might have repelled flying mosquitoes prior to first contact was investigated by comparing responses to sticky-net with untreated control. The set-up was identical to the previous experiment, but here contact with the sticky-netting was prevented by an outer barrier of untreated net (Figure 2B). Two layers of untreated netting were used in controls. For 30 min, all contacts were recorded as ‘visit’ events unless the mosquito landed on the net (defined as a contact of approximately more than one second), when a ‘rest’ event was recorded. In total, 72 individual mosquitoes were monitored on six human volunteers. The numbers and duration of contact and rest events were quantified in time-event logs generated by the Noldus event recorder.

### Data analyses

Data were assimilated in Microsoft Excel and analysed using non-parametric statistical methods in Stats Direct software V.2.4.5. Random effects regression analysis (RERA) and random effects cross-sectional regression analysis (CSRA) were done using Intercooled Stata software V.8.0.

## Results

### Semi-field tests with a human-baited sticky-net

**Figure 3:**
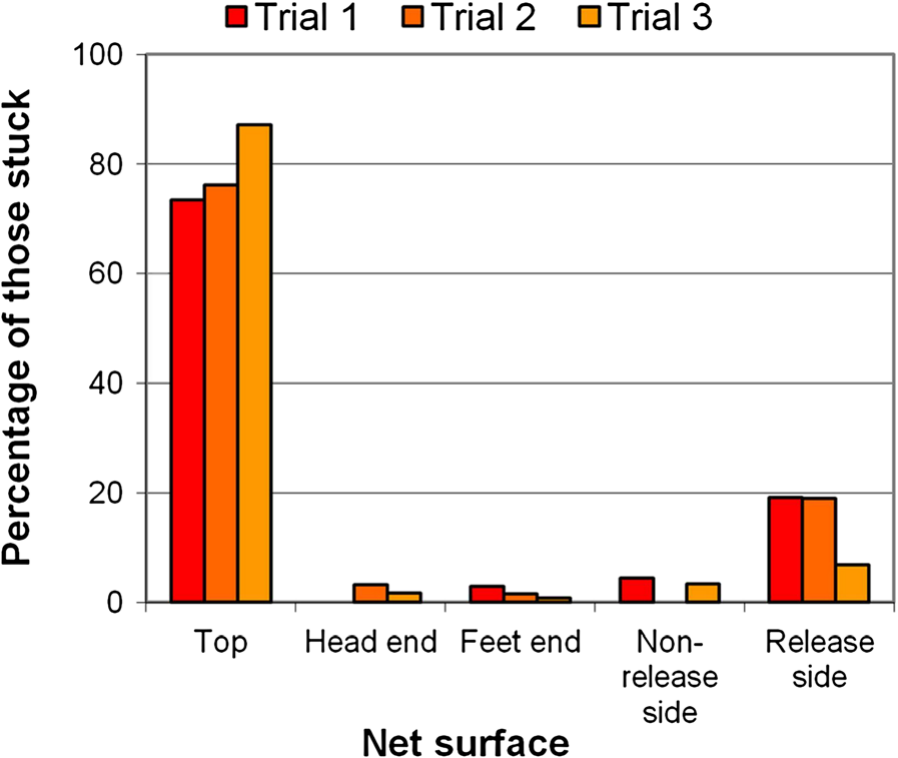
**Results from semi-field tests with a human-baited sticky-net.** The percentage of *An. gambiae* caught on each surface of the net (expressed as a percentage of total mosquitoes caught by the net) for all three experiments. The difference between the numbers on the top net surface and the other sides was significant in all three experiments (χ^2^ =101.5, 104.7, 268.0 in trials 1, 2 and 3 respectively; p < 0.001).

**Figure 4:**
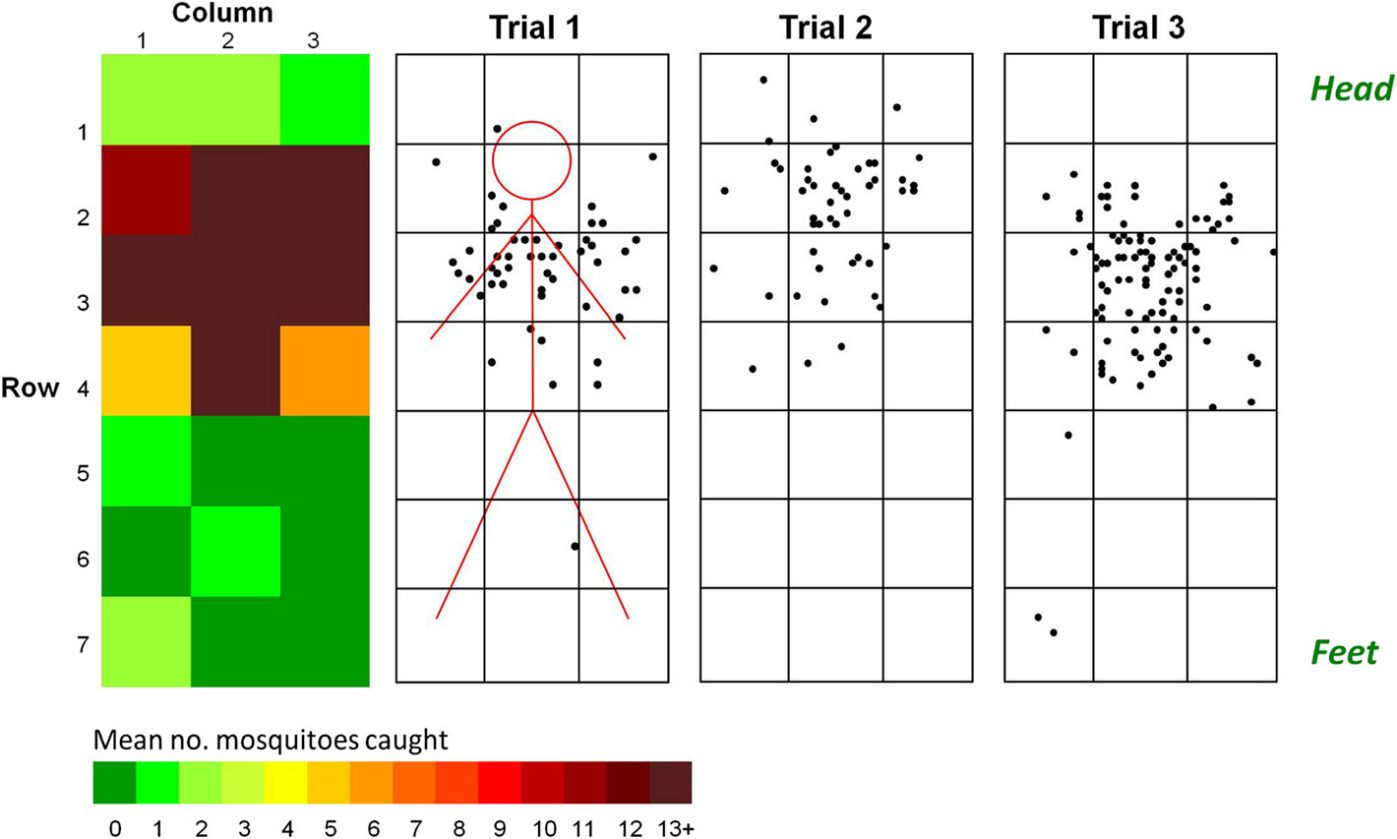
**Location of captured mosquitoes on the top surface of a human-baited sticky-net in semi-field experiments.** Diagram of results from semi-field tests with a human-baited sticky-net, showing the positions of all individual mosquitoes within each 30 sq cm square on the top surface of the net, for each experimental repeat. The coloured figure on the left shows the sum of all three experiments, in each 30 sq cm square. The numbers caught in rows 2, 3 and 4 alone (70% of the total) are significantly greater than expected (χ^2^ = 119.1, 99.3 and 164.5 for each experiment respectively; p < 0.001).

In three trials, 81% of all *An. gambiae* released were caught by the sticky-net. A significantly higher proportion of mosquitoes were caught on the top surface of the net after adjustment for area (74, 76, 87%, respectively in each trial) than on the sides (χ^2^ =101.5, 104.7, 268.0, respectively; p < 0.001) (Figure 3). Examination of the exact location of each mosquito captured on the top surface of the net (Figure 4) showed that nearly 70% of the mosquitoes caught on this surface were within a 90 × 90 cm area over the head and chest, a distribution pattern that was highly significant (χ^2^ = 119.1, 99.3, 164.5 for each experiment respectively; p < 0.001).

### Laboratory tests with a human-baited sticky-net

**Figure 5:**
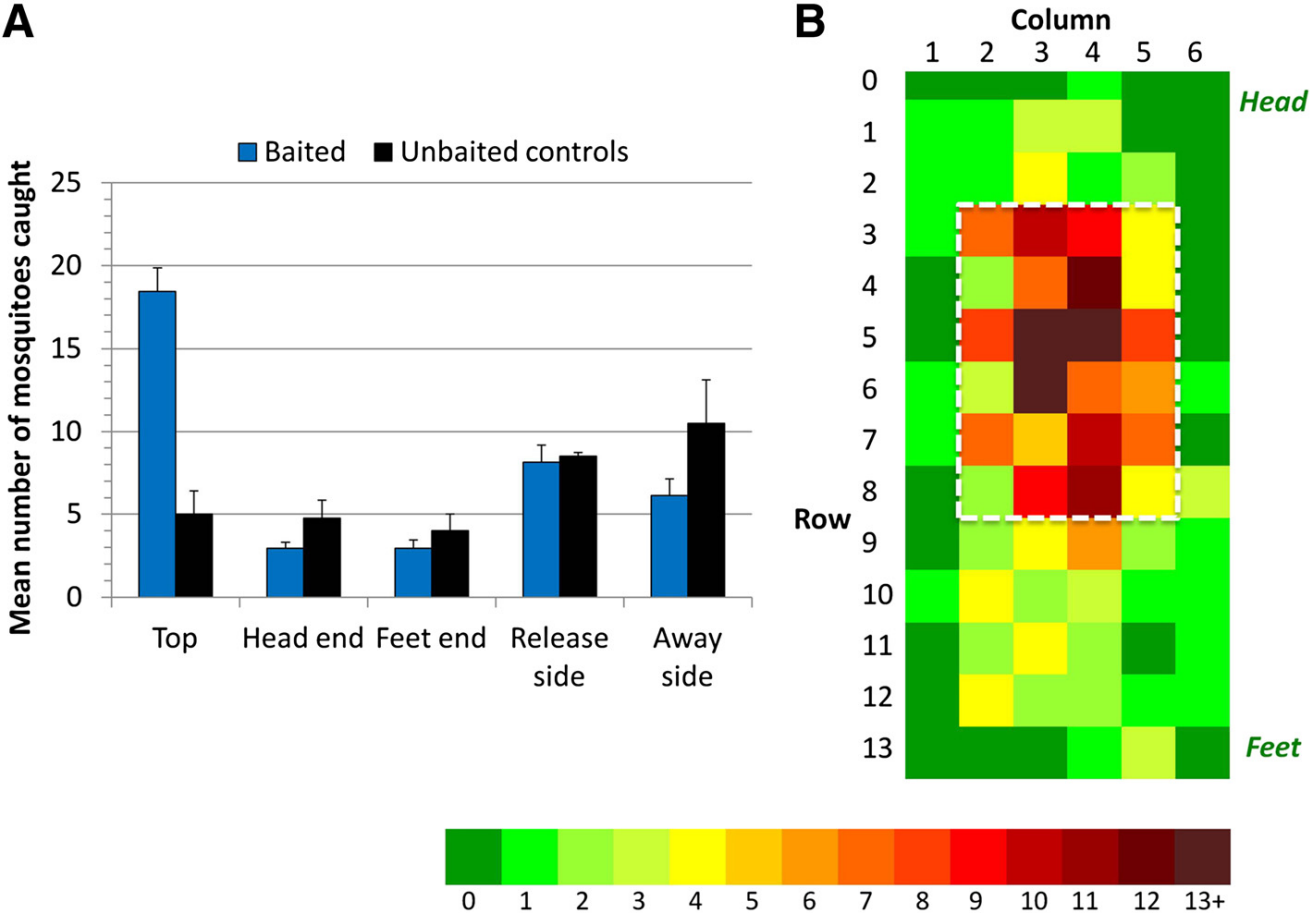
**Results of human-baited rectangular sticky-net from 14 laboratory experiments with seven human volunteers. A**. Mean (± SE) number of mosquitoes caught on each surface of the rectangular sticky-net in laboratory trials for human-baited and unbaited controls. Significantly more mosquitoes were caught on the top of the net than on the sides (after adjusting for area) in human-baited tests only (χ^2^ =192.7, p < 0.001). In unbaited nets, significantly more mosquitoes were caught on the sides than on the top (χ^2^ = 9.6; p = 0.047). **B**. Density distribution of caught mosquitoes each 15 sq cm square on the top surface of the net for all 14 experiments. Data are arranged such that in all experiments, the head end is labelled row 0, and the mosquito release side is labelled column 1. The white rectangle shows the 60 × 90 cm area (30% of the total top surface) within which 70% of all mosquitoes on the top surface were caught.

The mean capture rates of 38.6 and 32.8% in human-baited trials (14 trials, SD = 10.3, range 23-59%) and unbaited control trials (four trials, SD = 9.9, range 22-43%) were not significantly different (CSRA, p = 0.297). However, significantly more mosquitoes were caught on the top of the net than on the sides (after adjusting for area) in all human-baited tests (χ^2^ = 192.7, p < 0.001). This was significantly different to the unbaited controls (CSRA, p < 0.001), where the highest proportions of captured mosquitoes were on the sides of the net (χ^2^ = 9.6; p = 0.047) (Figure 5A).

As in the semi-field trials, the distribution of mosquitoes on the top surface of baited nets was not random (χ^2^ = 103.7; p <0.001): over 70% of those captured were within the 60 × 90 cm area above the sleeper’s head and chest, equivalent to 30% of the bed net’s top surface area (Figure 5B).

There were no significant differences between capture rates at release and non-release sides, or at sides A and B, or between the vertical end surfaces (RERA, p = 0.325), even when the orientation of the person was accounted for (RERA, p = 1.0). Mosquito release position did not affect the numbers that were caught on the top surface whether released high/low, on release/opposite side, or side A/B (RERA, p = 0.441, p = 0.387 and p = 0.411 respectively).

### Reliability and accuracy of the sticky-net

#### Laboratory tests with a human-baited pitched sticky-net

**Figure 6:**
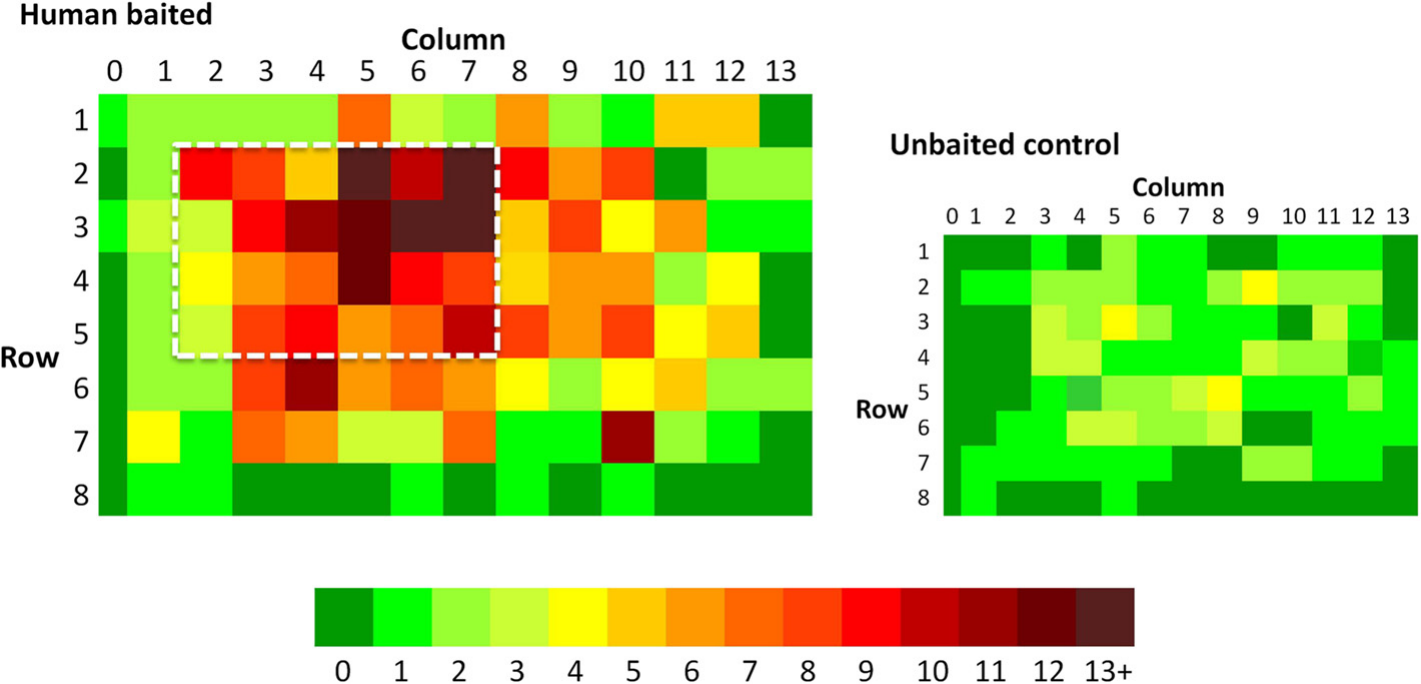
**Density distribution of mosquitoes caught on the side surfaces of human-baited and unbaited control pitched nets.** Data for both sides of the net are combined. Column numbers are shown relative to the sleeper’s orientation (i e*,* column 0 is always at the head). The hatched white rectangle in the human-baited net delineates the area where 44% of all mosquitoes were caught.

The mean capture rate in 16 trials was 32.6% (SD = 9.5, range = 18-46%), significantly more than the unbaited controls (mean = 17.3%; SD = 8.3; range = 10-33%) (RERA, p < 0.001) and similar to capture rates with the rectangular net (38.6%; RERA, p = 0 · 107). As no significant differences were seen in numbers captured on either side of the net or relative to release position, data at equivalent positions on both sides were combined for analysis. Distribution of mosquitoes in the baited net was not random (Figure 6) but differed significantly between rows and between columns (χ^2^, p < 0.001). As in the previous experiments, clustering occurred over the sleeper’s head and torso (rows 2–5, columns 2–7), such that 44% of captured mosquitoes were found within this 22% of the pitched surface area. No such pattern was seen with unbaited controls.

#### Direct observation of the effect of sticky-net surface orientation

**Figure 7:**
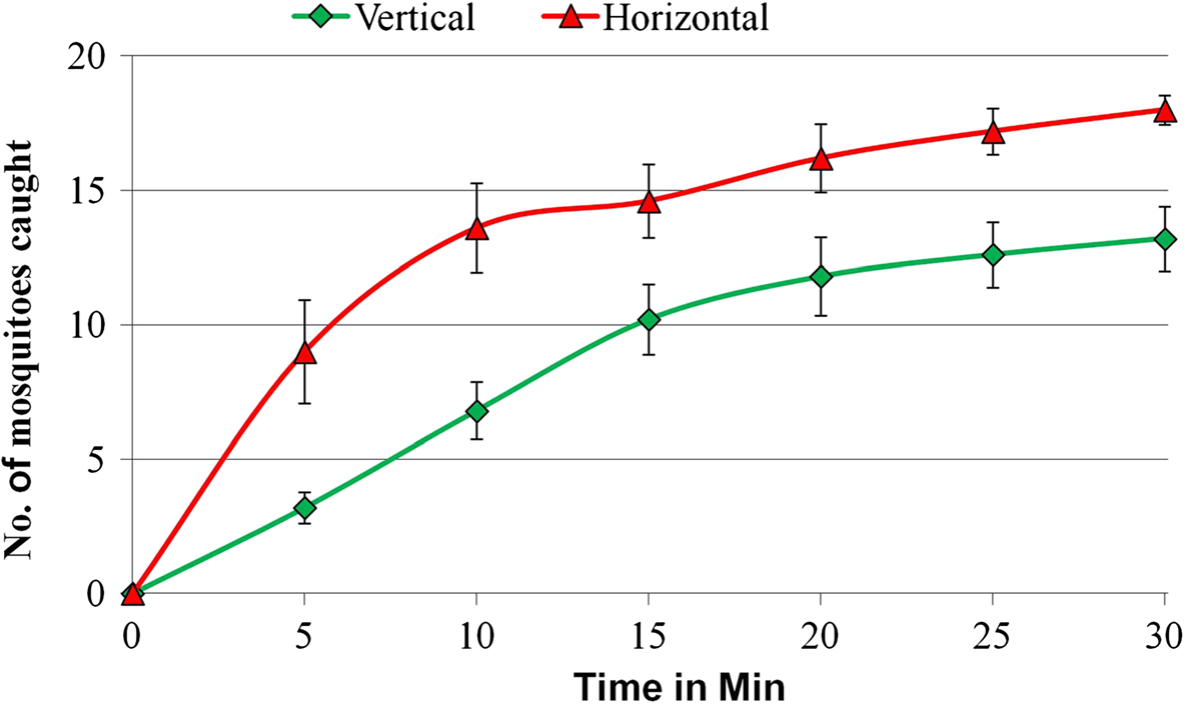
**Cumulative totals of mosquitoes captured at vertical and horizontal surfaces at five-minute intervals.** Cumulative totals are from an initial total of 20 released mosquitoes. N = 5 for both vertical and horizontal surfaces. Standard errors are shown.

In experiments with groups of mosquitoes (Figure 7), visit rates at horizontal surfaces were slightly but not significantly greater than at vertical surfaces (Mann–Whitney, p = 0.0635), but capture rates (S-R) at horizontal surfaces were higher (Mann–Whitney, p = 0.0079) (Table 1).

**Table 1:** Frequencies of behavioural events observed in groups of 20 mosquitoes at vertical and horizontal sticky-nets

	**Mean no of visits (V)**	**Mean no stuck (S)**	**Mean no of contacts (S + V)**	**Mean no captured (S-E)**
**Vertical**	84.4 (SD = 31.6)	15.6 (SD = 3.4)	100.0 (SD = 31.7)	13.2 (SD = 2.7)
**Horizontal**	135.2 (SD = 39.8)	21.2 (SD = 1.5)	156.4 (SD = 39.5)	18.0 (SD = 1.2)
**Vertical vs. horizontal Mann–Whitney**	p = 0.065	p = 0.0238	p = 0.0556	p = 0.0079

**V** = total number of visits to the net that did not result in capture; **S** = total number of mosquitoes that were captured;

**E** = number of mosquitoes that escaped from the net surface after temporary capture.

In individual mosquito tests, the mean time to capture was significantly lower at a horizontal surface (429 sec, SD = 323) than a vertical surface (665 sec, SD = 459) (simple cross-sectional regression analysis, p = 0.01), but significantly more contacts were needed at horizontal (mean = 21.5, SD = 12.7) than at vertical surfaces (mean = 14.6, SD = 7.9) (negative binomial regression analysis, p = 0.024; incident rate ratio = 1.34) before mosquitoes were captured.

In summary, these data suggest that horizontal surfaces were less effective at capturing mosquitoes, although they were visited as frequently as vertical surfaces.

#### Repellency of the adhesive treatment

The mean number of visits at a sticky net (mean 833; SD = 529) was lower than, but not significantly different to, untreated controls (mean 1,220; SD = 768), (negative binomial regression analysis p = 0.065, incident rate ratio, 1.29). The mean total time spent resting at sticky-net surfaces (mean = 369 sec; SD = 244) was lower than at untreated controls (446 sec; SD = 368) although again, not significantly so (simple linear cross sectional regression analysis, p = 0.283). The results suggested that any repellent effects of the adhesive were mild and unlikely to have influenced the results recorded.

## Discussion

These are the first experimental data describing the distribution of mosquito activity at a human-occupied bed net. Using a simple technique, results demonstrated that the majority of *An. gambiae* approach the host and land at the top surface of a human-baited bed net, and that this activity is localized within a relatively discrete area directly above the torso and head of the bed net occupant. The observed behaviour was consistent across studies carried out in two laboratories, with different experimental set-ups, and with multiple human volunteers.

While bias arising from the experimental procedures was a concern, especially the possibility that the horizontal top surface might have caught disproportionately more mosquitoes than the vertical side surfaces, three key findings indicated that this was unlikely. First, in the absence of host bait, significantly fewer mosquitoes were caught on the top surface of a standard net (Figure 5A). Second, the pitched net experiment eliminated any potential influence of surface orientation yet revealed a behaviour pattern equivalent to that on standard nets. Third, observation studies indicated that the number and frequency of visits were similar at both vertical and horizontal surfaces (Table 1) and that horizontal surfaces were less effective in capturing mosquitoes. The fact that significantly more horizontal than vertical contacts were needed before capture occurred, suggests the results might even have underestimated activity at the top of the net.

It is proposed therefore that the activity described is a true representation of *An. gambiae* behaviour. As such, this provides evidence for the earlier proposition that mosquitoes are attracted to the top surface of a bed net by a hypothetical rising plume of putative attractants emanating from the prone human sleeper [15] and funnelled upwards by the net itself [9]. The exact source of these attractants remains elusive. Studies with seated human hosts suggested that foot odour was a key attractant for *An. gambiae*[16], though Dekker *et al.* later showed that when the subject was lying down with the legs raised, significantly fewer mosquitoes bit the legs and feet compared to the rest of the body [15]. More recent studies suggested that body sweat was attractive [17-19] while breath had both attractant and repellent components [20]. Since nothing is known of how these different odours disperse or mix after emission by a prone human, it is impossible to draw conclusions as to which is the most important cue from the results presented here. Nonetheless, the remarkable ‘focus’ of behavioural activity within a discrete area directly over the sleeper’s head and torso has never been reported previously and offers potential for further exploration of host attractants as well as for exploitation in the design of LLINs.

Interestingly, mosquito release height or location did not significantly affect the distribution of mosquitoes caught on the sticky-nets, suggesting that arrival at the host by *An. gambiae* might be similar whether mosquitoes enter through windows, doors or eaves*.* However, the sticky net’s inability to capture mosquitoes on first contact clearly was a limitation because more detailed investigation of arrival patterns and foraging behaviour at the bed net-human interface were not possible. Further work is needed to investigate these important events.

A next step from this study is to measure responses of this and other mosquito species to commercially available LLINs, to determine if arrival patterns are similar on the various insecticide-treated bed nets currently available. This is particularly important for two-in-one type nets. For example, where pyrethroid-treated side netting is combined with a non-pyrethroid treated top surface: preferential contact with the top surface would expose vectors to the non-pyrethroid (the desired effect); but if activity predominated at the sides, two-in-one nets would perform no differently to existing LLINs. In a worst-case scenario, infrequent, limited, sublethal dose exposure to the insecticide on top might even promote resistance.

## Conclusions

The results indicated that *An. gambiae* host-seeking activity occurred predominantly within a key area on the top surface of a protective bed net, directly above the sleeper’s head and chest. The implications and full potential of this result may not be immediately obvious, but as shown by the remarkable successes with tsetse flies [21,22], exploration of basic vector behaviour can lead to novel tools that address current and future challenges in malaria vector control [23,24].

## Abbreviations

CDC: Centers for disease control; CSRA: Random effects cross-sectional regression analysis; ITN: Insecticide-treated net; KEMRI: Kenya medical research institute; LLIN: Long-lasting insecticidal net; LSTM: Liverpool school of tropical medicine; RERA: Random effects regression analysis; RH: Relative humidity; SD: Standard deviation.

## Competing interests

The authors declare that they have no competing interests.

## Authors’ contributions

AL and PJM conceived and designed the experiment. AL performed the experiment and analysed the data. AL and PJM wrote the paper. All authors read and approved the final manuscript.
